# The widespread biting midge *Culicoides impunctatus* (Ceratopogonidae) is susceptible to infection with numerous *Haemoproteus* (Haemoproteidae) species

**DOI:** 10.1186/s13071-017-2317-z

**Published:** 2017-08-25

**Authors:** Rita Žiegytė, Mikhail Yu. Markovets, Rasa Bernotienė, Andrey Mukhin, Tatjana A. Iezhova, Gediminas Valkiūnas, Vaidas Palinauskas

**Affiliations:** 1 Nature Research Centre, Akademijos 2, 21, LT-09412 Vilnius, Lithuania; 2 Russian Academy of Sciences, Biological Station Rybachy of the Zoological Institute, Rybachy, 238535 Kaliningrad Region Russia

**Keywords:** Sporogony, *Haemoproteus*, Birds, *Culicoides impunctatus*, Vectors, Transmission

## Abstract

**Background:**

*Haemoproteus* parasites are widespread, and some species cause disease in wild and domestic birds. However, the insect vectors remain unknown for the majority of species and genetic lineages of avian *Haemoproteus*. This information is crucial for better understanding the biology of haemoproteids, the epidemiology of haemoproteosis, and the development of morphological characters of sporogonic stages in wildlife haemosporidian parasites. It remains unclear whether the specificity of *Haemoproteus* parasites for vectors is broad or the transmission of a given parasite can be restricted to a single or few species of vectors. The aim of this study was to examine the sporogonic development of four species of common European avian haemoproteids in the common biting midge *Culicoides impunctatus.*

**Methods:**

Wild-caught females of *C. impunctatus* were infected experimentally by allowing them to take blood meals on naturally infected *Muscicapa striata*, *Cyanistes caeruleus*, *Ficedula hypoleuca* and *Motacilla flava* harbouring mature gametocytes of *Haemoproteus balmorali* (genetic lineage hSFC9), *H*. *majoris* (hPARUS1), *H*. *motacillae* (hYWT1) and *H. pallidus* (hPFC1), respectively. Infected insects were collected, maintained under laboratory conditions and dissected daily in order to detect the development of ookinetes, oocysts and sporozoites. Microscopic examination and polymerase chain reaction based methods were used to detect the parasites. Bayesian analysis was applied to identify phylogenetic relationships among *Haemoproteus* lineages.

**Results:**

All investigated parasites completed sporogony in *C. impunctatus*, indicating broad susceptibility of this biting midge for numerous *Haemoproteus* parasites. Ookinetes, oocysts and sporozoites were reported, described and compared morphologically. The investigated parasite species can be distinguished at the sporogony stage, particularly with regards to the morphology and rate of development of mature ookinetes. Analysis of data from the literature, and this study, shows that 12 genetically distantly related *Haemoproteus* parasites complete sporogony in *C. impunctatus*.

**Conclusions:**

Susceptibility of *C. impunctatus* is broad for *Haemoproteus* parasites, indicating that this biting midge is an important natural vector of numerous species of avian haemoproteids in Europe. Some *Haemoproteus* species can be readily distinguished using morphological characters of ookinetes and sporozoites, as well as the rate of ookinete development. These characters can be used for the identification of *Haemoproteus* species during sporogony in vectors, and are worth more attention in these parasite taxonomy studies at the species levels.

**Electronic supplementary material:**

The online version of this article (doi:10.1186/s13071-017-2317-z) contains supplementary material, which is available to authorized users.

## Background

Species of *Haemoproteus* are widespread and infect birds throughout the world [1–4]. Recent molecular studies have revealed the great diversity of haemosporidian parasites [5, 6]; however, the insect vector species of the majority of avian *Haemoproteus* parasites as well as their role in transmission remain unknown [2, 7]. Information about the sporogonic development of over 90% of the described avian *Haemoproteus* species is absent or remains fragmentary [1, 8]. Experimental studies on the transmission of *Haemoproteus* species by biting midges are difficult to design, because of the difficulties to infect, rear and dissect these tiny insects.

Currently used PCR-based tools markedly increased the determination of significant links between parasite lineages and blood-sucking insects [9–11]. However, this methodology does not distinguish invasive (sporozoites) and non-invasive (blood forms, ookinetes and oocysts) stages, and thus cannot provide conclusive evidence regarding whether the PCR-positive insects can act as vectors. In other words, the results of solely molecular diagnostics indicate only the possibility as natural vectors [8, 12–15]. Many recent studies used solely PCR-based tools in vector studies of *Haemoproteus* [9, 16, 17] and *Plasmodium* [10, 13, 18, 19] parasites in wild-caught blood-sucking dipteran insects. Results of these studies simplify the search for possible vectors due to a narrowing of the range of potential blood-sucking insects, which might act as vectors. However, the detection of infective sporozoites is required to prove that a given insect species can act as a vector. This is a particularly sensitive issue, due to the possibility of long-lasting abortive sporogonic development of *Haemoproteus* parasites in resistant insects. Recent experimental studies show that PCR-based diagnostics should be used carefully in vector studies because they can detect DNA of parasites in insects for several weeks after the initial infection. Moreover, they do not identify abortive sporogonic development and thus cannot prove if the insects are competent vectors of the parasites [11].

Research on haemoproteids and their vectors is important because some *Haemoproteus* parasites have been reported to cause disease and even lethal pathology in non-adapted avian hosts [20–26]. Moreover, intense infections of these parasites are virulent to the blood-sucking insects and can cause their mortality [27–30]. Only few experimental studies have focused on the transmission of *Haemoproteus* parasites in wildlife [1, 31, 32], posing an obstacle to understanding the epidemiology of diseases caused by these infections and the patterns of their distribution in wildlife. The available information about sporogony of *Haemoproteus* species and knowledge on their transmission and specificity for blood-sucking insects is currently insufficient.

Recently, a simple methodology of experimental infection of wild-caught *Culicoides* biting midges with *Haemoproteus* parasites was developed [1], and the sporogony of nine *Haemoproteus* species was investigated in *Culicoides impunctatus* biting midges [14, 15, 27, 33]. We predicted that many other common avian haemoproteids can be transmitted by this insect. To test this hypothesis, we examined the sporogony of four additional *Haemoproteus* parasites belonging to the subgenus *Parahaemoproteus*, *Haemoproteus balmorali* (cytochrome *b* genetic lineage hSFC9), *Haemoproteus majoris* (hPARUS1), *Haemoproteus motacillae* (hYWT1) and *Haemoproteus pallidus* (hPFC1) using the same methodology. These haemoproteid infections are widespread in Europe [1, 34]. The results have provided an opportunity for broad comparative research. The aim of this study was to follow sporogony of these parasites in *C. impunctatus*, which is widespread in Europe and willingly takes avian blood meals [33, 35]. Additionally, we reviewed previous studies addressing sporogonic development of *Haemoproteus* species in the same insect species, and provide comparative analysis of the morphological features of sporogonic stages of investigated parasites.

## Methods

### Collection of material and microscopic examination

This study was carried out at the Biological Station “Rybachy” of the Zoological Institute of the Russian Academy of Sciences on the Curonian Spit of the Baltic Sea (55°15’N, 20°86’E) between 23rd May and 29th June in 2015. Birds were captured by mist nets, stationary funnel traps [36] and nest box traps [37].

About 50 μl of blood was taken in heparinized microcapillaries by puncturing the brachial vein. A small drop was used for preparation of two blood smears from each individual. Residual blood was stored in SET-buffer for molecular analysis [38]. The smears were air-dried, fixed in absolute methanol and stained with Giemsa stain solution, as described by Valkiūnas et al. [39]. Approximately 100–150 fields were examined at low magnification (400×), and then at least 100 fields were studied at high magnification (1000×). Intensity of parasitemia was estimated as a percentage by actual counting of the number of parasites per 1000 erythrocytes or per 10,000 erythrocytes if infections were light (< 0.1%), as recommended by Godfrey et al. [40]. *Haemoproteus* parasites were identified according to Valkiūnas [1] and Dimitrov et al. [34]. Naturally infected birds with single infections of corresponding parasite species were kept indoors in a vector-free room under controlled conditions [20 ± 1 °C, 50–60% relative humidity (RH), the natural light-dark (L/D) photoperiod] and fed with standard diet for seed-eating or insectivorous bird species. These birds were used as donors to infect biting midges. All birds survived until the end of this study and were released in the same area as captured after experiments.

### Sampling and experimental infections of biting midges

Experimental infection of biting midges with *Haemoproteus* parasites was performed near Lake Chaika, located close to the village Rybachy, where the density of biting midges is high [41]. All experimental infections were performed between 10th and 20th June when the first generation of *C. impunctatus* predominate [42]. Unfed flies (wild-caught controls) were collected by entomological nets at the same study site before experiments. Approximately 100 wild-caught females were sampled, fixed in 70% ethanol and used for species identification, which was done using morphological characters [43]. Possible natural infection by *Haemoproteus* parasites in the wild-caught insects was tested using PCR-based methods (see description below).

Wild *C. impunctatus* females were infected by allowing them to take blood meals on four naturally infected birds harbouring single infections of different species of *Haemoproteus* parasites [1, 14]. We used one individual of each spotted flycatcher *Muscicapa striata*, blue tit *Cyanistes caeruleus*, pied flycatcher *Ficedula hypoleuca* and blue-headed wagtail *Motacilla flava* harbouring mature gametocytes of *H*. *balmorali* (hSFC9), *H*. *majoris* (hPARUS1), *H*. *motacillae* (hYWT1) and *H. pallidus* (hPFC1), respectively. Low level parasitemia (< 1%) was present in all donor birds throughout the course of the experiment. Each bird was used 2–3 times for infection of midges. One uninfected common crossbill *Loxia curvirostra* was used to feed a control group of biting midges during experiments. Birds were hand held using rubber gloves. Biting midges were allowed to feed on the head of naturally infected bird for approximately 15 min. *Culicoides impunctatus* females willingly took blood meals on the feather-free region of bird’s head. Once several flies started to feed on a bird’s head, it was inserted into an insect cage. The cages (12 × 12 × 12 cm) were made of fine-mesh bolting silk. A zip fastener was sewn into one wall of the cage to permit entry of the bird’s head. Engorged midges flew off the bird’s head on the wall of the insect cage, which was then closed. The cages with engorged flies were transported to the laboratory and held at temperatures ranging between 14 °C (at night) and 24 °C (during the day), 60 ± 2% RH and photoperiod of 17:7 h L/D).

Midges were supplied with 5–10% saccharose solution; pads of cotton wool moistened in this solution were placed on the top of each insect cage. Eighty-two *C. impunctatus* biting midges infected with *H*. *balmorali* (*n* = 17 infected insects), *H*. *majoris* (*n* = 20), *H*. *motacillae* (*n* = 19) and *H. pallidus* (*n* = 26) were dissected for detection of ookinetes, oocysts and sporozoites. Twenty engorged *C. impunctatus* biting midges were used as controls during experiments.

### Dissection of biting midges and preparation of ookinetes, oocysts and sporozoites specimens

Details of the dissection and staining methods of ookinetes, oocysts and sporozoites were described in [1, 14, 44]. Briefly, infected females were anesthetized by placing them into a tube with a cotton pad wetted with 96% ethanol and closed for several minutes. Dissection was performed under the binocular stereoscopic microscope Olympus SZX10 (Tokyo, Japan). To eliminate contamination of samples, we used a new dissecting needle for each dissected biting midge.

We examined midgut contents for ookinetes between 1 h post-infection (hpi) and 4 days post-infection (dpi), midgut wall for oocysts 2–5 dpi, and salivary glands for sporozoites 5–12 dpi. Content of the midgut was extracted by cutting a terminal segment of the abdomen and pressing the content out on the glass slide. The midgut content was mixed with drop of normal saline and a thin smear was prepared; the smears were dried in the air, fixed with methanol, and stained with 10% Giemsa stain, in the same way as blood films.

Oocysts were visualised by adding a minute drop of 2% mercurochrome solution on freshly prepared midgut preparation, which was then covered with a coverslip. Midguts with oocysts were fixed in 10% formalin solution and then in 70% ethanol for permanent preparations [1]. Thereafter midgut preparations were washed with distilled water, stained with Ehrlich’s haematoxylin, steeped in water containing a 0.45 g of sodium bicarbonate and differentiated with acid ethanol and then again steeped in water containing sodium bicarbonate. Then, each preparation was dehydrated with 70 and 96% ethanol, cleared by putting a drop of clove oil and xylene over the preparation, and finally mounted in Canada balsam, covered with a cover slip and dried in the air for approximately 1 week.

Preparations of sporozoites were made after extraction of salivary glands from biting midges. Insects head and thorax were placed in a small drop of saline. Salivary glands were gently pressed out from the thorax, crushed using a needle and mixed with a tiny drop of saline. Preparations were dried in the air, fixed with absolute methanol, and stained with 4% Giemsa stain. After dissection, all residual parts of insects were placed in 96% ethanol for PCR-based confirmation of the parasite lineages and insect species (see description below).

Representative preparations of blood stages (accession numbers 48,948–48951NS), ookinetes (48952–48955NS), oocysts (48956–48958NS) and sporozoites (48959–48962NS) were deposited in Nature Research Centre, Vilnius, Lithuania. The statistical analyses of parasites sporogonic stages were carried out using the “Statistica 7” package. Student’s t-test for independent samples was used to determine statistical significance between mean linear parameters of parasite features. A *P*-value < 0.05 was considered significant.

### DNA extraction, PCR and sequencing

The total DNA was extracted from all samples (bird blood and biting midges) using an ammonium acetate extraction method [45]. For the parasite lineage determination, we used a nested PCR protocol [39, 46]. A segment of parasite mitochondrial *cytb* gene fragment was amplified using initial primers HAEMNFI and HAEMNR3 which amplify fragments of *cytb* gene of haemosporidians belonging to genera *Haemoproteus*, *Plasmodium* and *Leucocytozoon*. For the second PCR, we used HAEMF and HAEMR2 primers, which are specific to *Haemoproteus* and *Plasmodium* spp. All amplification procedures were in accord to the protocols by [38, 46]. Additionally, we used primers reported by Beadell et al. [47] for *cytb* gene fragment amplification from investigated bird blood to confirm that birds were infected by a single *Haemoproteus* infection. The amplification was evaluated by running 1.5 μl of the final PCR product on a 2% agarose gel. One negative control (nuclease-free water), one positive control (*Haemoproteus* spp. microscopy positive blood sample in the case of blood testing, and thorax of biting midge experimentally infected with *Haemoproteus belopolskyi*, in the case of biting midge testing) were used per every 8 samples to control for false amplifications. Mixed infections were determined both by the microscopic examination of blood films and the visualization of double-base calling in sequence electropherograms.

Fragments of DNA from all positive samples were sequenced from the 5′- end with the primer HAEMF using dye terminator cycle sequencing (big dye) and loaded on an ABI PRISM 3100 capillary sequencing robot (Applied Biosystems, Foster City, California) [46]. The “Basic Local Alignment Search Tool” (National Centre for Biotechnology Information website: http://www.ncbi.nlm.nih.gov/BLAST) was used to determine genetic lineages of detected DNA sequences, which were deposited in GenBank (KY451714–KY451721).

DNA extracted from individual biting midges was used to confirm the identification of *C. impunctatus* used in our experiments. We applied the insect specific primers LCO149 and HCO2198 to amplify a fragment of cytochrome *c* oxidase subunit 1 (*cox*1) of mitochondrial DNA [48]. Sequences corresponded to the *C. impunctatus* DNA sequences available in GenBank (KY627800).

### Phylogenetic analysis

To define the phylogenetic placement of obtained lineages, we used amplified *cytb* sequences (478 bp fragments) and available DNA fragments from the MalAvi database (http://mbio-serv2.mbioekol.lu.se/Malavi/). Bayesian phylogeny was constructed using MrBayes version 3.1.2 [49]. We used the General Time Reversible Model including rate variation among sites (GTR + I + G), as selected model of sequence evolution suggested by the mrModeltest 2v software [50]. The analysis was run for a total of 8 million generations with a sample frequency every 100 generations. Before constructing a majority consensus tree, 25% of the initial trees in each run were discarded as “burn in” periods. The remaining trees were used to construct a Majority rule consensus tree. Phylogeny was visualized using Tree View 1.6.6 (http://evolution.genetics.washington.edu/phylip/software.html). The sequence divergence between lineages was calculated using a Jukes-Cantor model of substitution, with all substitutions weighted equally, implemented in the program MEGA 4 [51].

## Results

### Microscopic examination and PCR-based testing of wild-caught insects and birds

All biting midges used in experiment belonged to *C. impunctatus.* PCR-based analysis did not detect natural infections of *Haemoproteus* parasites in wild-caught biting midges, indicating that natural infection was unlikely in wild-caught experimental insects.

**Fig. 1:**
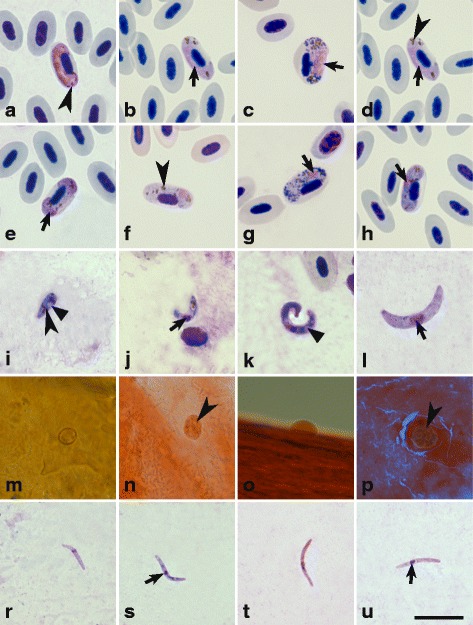
Mature microgametocytes (**a**-**d**), macrogametocytes (**e**-**h**), ookinetes (**i**-**l**), growing oocysts (**m**-**p**) and mature sporozoites (**r**-**u**) of *Haemoproteus balmorali* (**c**, **g**, **k**, **o** and **t**), *H*. *majoris* (**b**, **f**, **j**, **n** and **s**), *H*. *motacillae* (**d**, **h**, **l**, **p** and **u**) and *H. pallidus* (**a**, **e**, **i**, **m** and **r**). All images are from the methanol-fixed and Giemsa-stained thin films, except images **m**-**p**, which are from formalin-fixed whole mounts stained with Erlich’s haematoxylin. Short simple arrows: nuclei of parasites; arrowheads: pigment granules; triangle arrowheads: vacuoles. *Scale-bar*: 10 μm

Experimental birds (parasite donors) were naturally infected with single *Haemoproteus* infections: *M. striata* was parasitized by *H*. *balmorali* (lineage hSFC9) (Fig. 1c-g), *C. caeruleus* by *H*. *majoris* (hPARUS1) (Fig. 1b-f), *M. flava* by *H*. *motacillae* (hYWT1) (Fig. 1d-h) and *F. hypoleuca* by *H. pallidus* (hPFC1) (Fig. 1a-e). Both microscopy and two PCR assays revealed single *Haemoproteus* infections in all donor birds. No double-base calling in sequence electropherograms were detected.

### Sporogony research

Microscopic examination confirmed that *H*. *balmorali* (hSFC9) (Fig. 1c-t), *H*. *majoris* (hPARUS1, Fig. 1b-s), *H*. *motacillae* (hYWT1, Fig. 1d-u) and *H. pallidus* (hPFC1, Fig. 1a-r) completed sporogony in *C. impunctatus* biting midges*.* PCR and sequencing confirmed the presence of corresponding genetic lineages in experimentally infected *C. impunctatus* biting midges. The morphometric parameters of ookinetes, oocysts and sporozoites of all parasite species are given in Table 1.

**Table 1:** Morphometric parameters of ookinetes, oocysts and sporozoites of four *Haemoproteus* species in the biting midge *Culicoides impunctatus*. Measurements are given in micrometres. Minimum and maximum values are provided, followed in parentheses by the arithmetic mean and standard deviation

Feature	Measurements^a^			
	*H. pallidus*	*H*. *majoris*	*H*. *motacillae*	*H*. *balmorali*
Ookinete				
Length	7.5–9.6 (8.3 ± 0.5)	9.4–14.4 (12.1 ± 1.5)	11.1–20.0 (16.7 ± 2.5)	15.0–21.4 (18.0 ± 1.7)
Width	2.1–4.3 (3.0 ± 0.6)	1.4–3.0. (2.1 ± 0.4)	1.3–3.3 (2.3 ± 0.6)	1.4–3.0 (2.3 ± 0.4)
Area	12.3–24.8 (16.9 ± 3.3)	11.8–33.3 (19.5 ± 5.7)	20.2–47.4 (31.5 ± 8.8)	20.7–49.7 (32.3 ± 7.8)
Oocyst				
Min. diameter	2.7–4.6 (3.5 ± 0.6)	2.7–4.5 (3.6 ± 0.7)	3.5–6.1 (4.8 ± 1.0)	3.0–5.1 (4.2 ± 0.6)
Max. diameter	3.6–5.2 (4.3 ± 0.5)	3.6–6.0 (4.9 ± 0.8)	5.4–7.1 (6.3 ± 0.6)	3.8.1–6.9 (5.2 ± 0.8)
Area	7.3–16.0 (11.7 ± 2.5)	7.7–18.7 (14.5 ± 4.2)	17.4–33.4 (23.7 ± 6.0)	7.8–25.3 (17.7 ± 5.0)
Sporozoite				
Length	6.2–11.5 (8.9 ± 1.3)	6.6–13.5 (9.5 ± 1.5)	8.7–12.7 (10.2 ± 0.9)	7.7–12.0 (11.0 ± 1.0)
Width	0.6–1.4 (1.0 ± 0.2)	0.9–1.5 (1.1 ± 0.2)	0.7–1.7 (1.0 ± 0.1)	0.6–1.2 (0.9 ± 0.1)
Area	5.0–9.8 (7.3 ± 1.3)	5.7–13.2 (8.2 ± 2.0)	5.2–11.5 (8.2 ± 1.6)	5.0–9.5 (7.3 ± 1.1)

^a^Measurements of ookinetes (*n* = 21, methanol-fixed preparations 12–24 hpi), oocysts (*n* = 8–12, formalin-fixed preparations of mature parasites 2.5–4 dpi) and sporozoites (*n* = 21, methanol-fixed preparations 8 dpi)

Ookinetes of *H. pallidus* (Fig. 1i) developed quickly. Mature ookinetes of this parasite (Fig. 1i) were seen in the midgut contents of experimentally infected flies between 4 and 12 hpi. After 12 hpi, they were absent from midgut contents. This should be taken into consideration in future sporogony studies of this parasite. Fully grown ookinetes are carrot-like in form, with one end broader than another. The nucleus is located slightly off-centre (Fig. 1i). A small vacuole was visible near the nucleus in 40% of reported ookinetes. Pigment granules in ookinetes are located close to the narrower end, and rarely at the opposite end or close to both ookinete ends. Ookinetes of *H. pallidus* are significantly shorter than *H*. *majoris* (Student’s test, *t*_(40)_ = 10.87, *P* < 0.0001), *H*. *motacillae* (*t*_(40)_ = 15.03, *P* < 0.0001) and *H*. *balmorali* (*t*_(40)_ = 24.29, *P* < 0.0001) ookinetes (Table 1, Additional file 1: Table S1). Mean area of *H. pallidus* and *H*. *majoris* ookinetes does not differ significantly (*P* = 0.07), but area of *H. pallidus* ookinetes was significantly smaller than area of *H*. *motacillae* (*t*_(40)_ = 7.11) and *H*. *balmorali* (*t*_(40)_ = 8.34) ookinetes (both *P* < 0.0001).

Ookinetes of *H*. *majoris* (Fig. 1j) developed more slowly as compared to *H. pallidus*. They were seen between 12 and 24 hpi. Many large vacuoles were visible in the *H*. *majoris* ookinetes*.* Pigment granules are located in narrower end of young ookinetes (Fig. 1j). Fully developed ookinetes of *H*. *majoris*, *H*. *balmorali* and *H*. *motacillae* are elongate worm-like bodies, with the nucleus located slightly off-centre. The ookinetes of *H*. *balmorali* possess prominent vacuoles in the cytoplasm of the parasite (Fig. 1k). Ookinetes of *H*. *motacillae* (Fig. 1l) and *H*. *balmorali* (Fig. 1k) developed more slowly than those of *H. pallidus* and *H*. *majoris*; they were seen between 24 hpi and 1.5 dpi (Table 2).

**Table 2:** Species of *Haemoproteus* completing sporogony in the biting midge *Culicoides impunctatus*

*Haemoproteus* species	mtDNA lineage	Bird species	Report of sporogonic stage in experimentally infected females^a^			Reference
			Ookinetes	Oocysts	Sporozoites	
*H*. *balmorali*	–^b^	*Muscicapa striata*	1.5	3–4	5	[33]
*H*. *balmorali*	hSFC9	*Muscicapa striata*	1–1.5	4–6.5	6.5–10	This study
*H*. *belopolskyi*	hHIICT1	*Hippolais icterina*	1–3	3–6	7–12	[14]
*H*. *dolniki*	–	*Fringilla coelebs*	1.5	3–4	5	[33]
*H*. *fringillae*	–	*Fringilla coelebs*	1–2	3–4	5–8	[27]
*H*. *lanii*	–	*Lanius collurio*	1–2	3–4	5–8	[27]
*H*. *majoris*	hPARUS1	*Cyanistes caeruleus*	12–24^d^	2–4.5	6.5–7.5	This study
*H. minutus*	hTURDUS2	*Turdus merula*	1–4^d^	3–6	7–12	[14]
*H*. *motacillae*	hYWT1	*Motacilla flava*	1–1.5	3–4	5–8	This study
*H*. *noctuae*	hCIRCUM01	*Asio otus*	12^d^	–	7–9	[15]
*H*. *pallidus*	hPFC1	*Ficedula hypoleuca*	4–12^d^	1.5–2.5	6.5–7.5	This study
*H*. *parabelopolskyi* ^c^	–	*Sylvia atricapilla*	1–1.5	3	5–12	[27, 67]
*H*. *tartakovskyi*	–	*Loxia curvirostra*	1.5	3–4	5	[33]

^a^Time of sporogonic development is given in dpi, except several cases of ookinete development, which are given in hpi

^b^Data are absent

^c^Originally described as *Haemoproteus belopolskyi* [67]

^d^Hours post - infection

In different parasite species, oocysts were first seen in the midgut wall 2–4 dpi (Table 2), and they were reported in the midgut preparations 4–6 dpi (Fig. 1m-p). Oocysts appeared as small roundish bodies, and pigment granules were visible in some of them. In flattened midgut preparations, there was no significant difference in the mean area of oocysts (Table 1) among all parasites species (*P* > 0.05, Additional file 1: Table S2).

Sporozoites of *H. pallidus* (Table 1, Fig. 1r) and *H*. *majoris* (Fig. 1s) were reported in the salivary glands preparations between 6 and 7 dpi, and sporozoites of *H*. *motacillae* (Fig. 1u) and *H*. *balmorali* (Fig. 1t) between 5 and 10 dpi. Sporozoites of all species are fusiform bodies with slightly off-centre located nuclei and approximately equally pointed ends. There were no differences discernible in the length or area between sporozoites of *H. pallidus* and *H*. *majoris* and between *H*. *majoris* and *H*. *motacillae* (*P >* 0.05). However, the sporozoites of *H. pallidus* are significantly shorter (*t*_(40)_ = 3.83, *P* = 0.0004) and smaller (*t*_(40)_ = 2.06, *P* = 0.046) in area than those of *H*. *motacillae* and shorter (*t*_(40)_ = 4.84, *P* < 0.0001) than sporozoites of *H*. *balmorali* (Table 1). Sporozoites of *H*. *balmorali* are longer than sporozoites of *H*. *majoris* (*t*_(40)_ = 2.57, *P* = 0.014). Area of sporozoites significantly differs between *H*. *motacillae* and *H*. *balmorali* (*t*_(40)_ = 2.16, *P* = 0.037, Table 1, Additional file 1: Table S3).

### Phylogenetic analysis

**Fig. 2:**
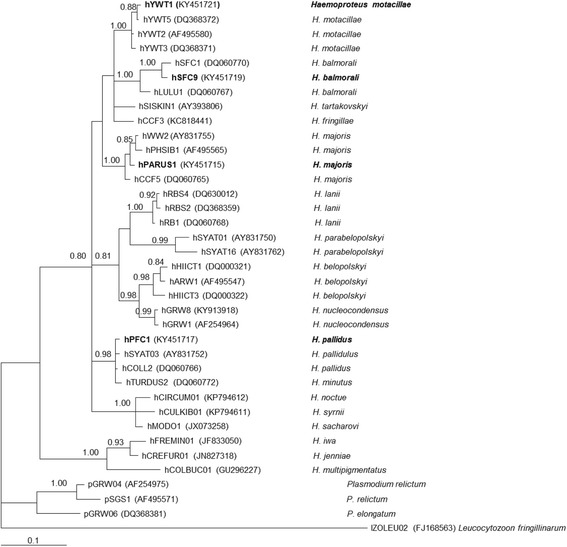
Bayesian phylogenetic tree of *cytb* gene sequences (379 nucleotides) of 33 *Haemoproteus* lineages and 3 *Plasmodium* lineages. One *Leucocytozoon fringillinarum* sequence was used as outgroup. Nodal support values indicate Bayesian posterior probabilities. The *scale bar* shows the expected substitutions per site. Lineage codes are given according to MalAvi database [5], followed by GenBank accession numbers in parentheses and parasite species names. *Haemoproteus* parasites used in this study are shown in bold

Bayesian analysis revealed that the investigated parasite lineages are distantly related (Fig. 2), with genetic divergence among them ranging between 4.3 and 6.4%. Three examined lineages cluster in separate well-defined monophyletic subclades representing morphologically characterized species; these are clades of several lineages belonging to *H*. *motacillae*, *H*. *balmorali* and *H*. *majoris* (Fig. 2). *Haemoproteus pallidus* (hPFC1) is phylogenetically the most distantly related lineage of the four parasites, and it is a representative of two morphologically characterized lineages of this morphospecies (Fig. 2). Phylogenetic analysis concurs with the marked morphological differences and patterns of ookinete development between *H. pallidus* and the three other examined *Haemoproteus* species.

## Discussion

The first studies on development of *Haemoproteus* parasites in *Culicoides* insects were carried out in the twentieth century [52–54]. They were reviewed by [1, 2, 8]. The studies on the development of haemoproteids in blood-sucking insects are few in comparison to numerous and detailed descriptions of these parasites in vertebrate hosts. In recent years, several studies provided detailed information about the sporogonic development of *Haemoproteus* spp. in vectors [1, 2, 8] however, information on this subject is crucial for better understanding the epidemiology of haemoproteosis [20, 22–24].

This study shows that complete sporogony of *H*. *balmorali* (hSFC9), *H*. *majoris* (hPARUS1), *H*. *motacillae* (hYWT1) and *H. pallidus* (hPFC1) occurs in the *C. impunctatus* biting midge, and these blood-sucking insects likely are natural vectors. Ideally, these results should be tested by transmission experiments using sporozoites developed in *C. impunctatus*. However, such experiments are difficult to perform due to the difficulties in obtaining and maintaining non-infected wild birds in captivity and permit obstacles in experimental research with wild birds. *Culicoides impunctatus* is common in Europe and is abundant at our study site [33, 41, 42, 55]. *Culicoides impunctatus* was often considered as mainly a mammalophilic species [56], but it willingly takes blood meal on birds [14, 15, 35, 41, 42]. According to former studies (Table 2), *C. impunctatus* is a competent vector of *Haemoproteus lanii*, *Haemoproteus fringillae*, *H*. *balmorali*, *Haemoproteus tartakovskyi*, *Haemoproteus dolniki*, *Haemoproteus parabelopolskyi* (genetic lineages of these parasites were not determined) and also *Haemoproteus minutus* (hTURDUS2), *Haemoproteus belopolskyi* (hHIICT1) and *Haemorpoteus noctuae* (hCIRCUM01). This study adds four *Haemoproteus* species, with their molecularly defined genetic lineages, to the list of parasites completing sporogony in *C. impunctatus* biting midges, indicating the susceptibility of this insect to numerous haemoproteid species (Table 2)*.* These insects are worth more attention in studies of the transmission and epidemiology of avian *Haemoproteus* spp. The role of other species of *Culicoides* in the transmission of *Haemoproteus* species remains unknown at the study site.

Recently, it was shown that *H*. *tartakovskyi* (hSISKIN1), *Haemoproteus syrnii* (hCULCIB01) and *H*. *noctuae* (hCIRCUM01) complete development in *Culicoides nubeculosus* biting midges [15, 57]. Former experimental studies indicated that *H*. *fringillae* develops sporozoites in *Culicoides crepuscularis*, *C*. *stilobezziodes* and *C. sphagnumensis* [2, 53]. Interestingly, *C. sphagnumensis* is a vector of 4 haemoproteids (*Haemoproteus mansoni*, *H*. *velans*, *H. fringillae* and *H*. *danilewskyi*) [2, 53, 58]. *Culicoides stilobezziodes* is a vector of 3 haemoproteids (*H*. *velans*, *H*. *fringillae* and *H*. *danilewskyi*) [53, 58, 59]. This is in accordance with results of this study, indicating susceptibility of the same *Culicoides* species to many *Haemoproteus* parasites.

Several studies have examined the in vitro gametogenesis and development of ookinetes in *Haemoproteus* species. Information about the development of haemoproteids both in vivo and in vitro is available for *H*. *fringillae*, *H. pallidus*, *H*. *parabelopolskyi*, *H*. *tartakovskyi*, *H*. *balmorali*, *H*. *dolniki*, *H*. *majoris*, *H*. *lanii*, *H. minutus* ([1, 60–62], this study) and *H*. *motacillae* (hYWT1) ([34], this study). The patterns of development of the same species in vivo and in vitro are similar, however some differences were recorded. For example, morphological differences during development of the same parasite species in vivo and in vitro were observed. Mainly, vacuoles were not reported in ookinetes of *H. pallidus* during in vitro development [60], but were often seen in this study. It is possible that these differences are due to differences of in vivo and in vitro conditions (midgut of vector vs artificial medium in a microtube). Development of *Haemoproteus* ookinetes can be readily initiated in vitro, however the results of such studies should be interpreted with caution, and some questions regarding the host-parasite interactions can be answered only using experiments performed in natural environments (vectors).

It is difficult to compare the rate of the parasites’ development using data from different studies because it depends markedly on temperature, which varied in the available studies. However, some patterns were reported. Available data show that *H. minutus*, *H. pallidus*, *H*. *majoris* and *H*. *noctuae* develop both in vivo and in vitro particularly fast, with mature ookinetes present within 0.5 dpi (Table 2). All other parasites tested developed mature ookinetes 1 dpi. This should be taken into consideration during haemoproteid vector research aiming to study mature ookinetes in midgut of insects.

The morphology of ookinetes differed in the four studied species (Fig. 1j- l). It appears that parasites which develop most rapidly have the smallest ookinetes. For example, ookinetes of *H. minutus* (hTURDUS2) [14] and *H. pallidus* (hPFC1) (this study) were exceptionally small (< 10 μm in length on average), and the time of their maturation in vivo is significantly faster (1–12 hpi) than of *H*. *majoris* (12 hpi) or *H*. *motacillae* ookinetes (24–36 hpi) (Table 2). The latter parasites have significantly larger mature ookinetes, in both length and area (Table 1) ([14], this study). Interestingly, *H. minutus* (hTURDUS2) and *H. pallidus* (hPFC1) are phylogenetically closely related parasites (Fig. 2), with a genetic divergence of only 0.9% in *cytb* gene. Small genetic differences between these two possibly relatively recently diverged parasites are in accordance with the similar morphology of their ookinetes and the rate of their ookinete development in comparison to other investigated species. It is thus possible that ookinetes of phylogenetically closely related haemosporidian species should have similar morphologies and rates of ookinete development. However, at present it is unclear to what extent phylogenetic differences are reflected by sporogonic stages; this needs more extensive research with phylogenetically closely and distantly related parasites.

A recent study by Nilsson et al. [63] used a multigene phylogenetic analysis to show that five *H*. *majoris cytb* lineages are closely related and cluster together, but likely represent distinct biological species. Mechanisms of reproductive isolation contributing to the maintenance of these closely related lineages at the same study site remain unclear, and they might be related to the different abilities and/or rates to develop in vectors. It worth mentioning that *H. pallidus* and *H*. *motacillae* complete sporogonic development in *C. impunctatus*, which can act as a vector at our study site. These parasites are prevalent in adult birds, but are extremely rare (*H. pallidus*) or absent (*H*. *motacillae*) in juveniles of the same bird species at our study site [1, 64]. Obstacles to the transmission of *H. pallidus* and *H*. *motacillae* remain unclear. *Culicoides impunctatus* biting midges are widespread at our study site and the period of their imago activity coincides with the birds’ breeding period [42]. The observed overlapping of vector activity and the appearance of birds’ offspring should favour transmission of infections to juvenile birds. However, this apparently does not happen, and the absence of some haemoproteids in juvenile birds raise questions about the presence of other important factors preventing transmission. For instance, there are no records of *H*. *motacillae* in juvenile yellow wagtails in northern Europe. At our study site, the absence of *H*. *motacillae* in juvenile birds can probably be explained by the different habitats occupied by vectors and the yellow wagtails. *Culicoides impunctatus* lives and breeds in moist forests [41], while the yellow wagtail inhabits meadows and wet pastures [65]. According to the literature [66], *C. impunctatus* midges only fly within 75 m from their breeding areas. Thus, differences in occupied habitats can separate insect and vertebrate hosts and act as an ecological barrier for transmission in breeding areas. Certainly, ecological factors require more attention in studies of transmission of haemosporidian parasites.

This study adds four haemoproteid species that complete sporogony in *C. impunctatus* and strengthen the published information, indicating that sporogony of *Haemoproteus* parasites is not restricted to a single species of biting midges [2]. Many *Haemoproteus* species can complete sporogonic development in the same species belonging to the genus *Culicoides* ([1, 2], this study). Our findings together with several other recent studies show that one species of biting midge is capable of transmitting many species of *Haemoproteus* ([1, 2, 8], this study). This conclusion is important epidemiologically. It could explain the high frequency of haemoproteid co-infections in some bird species, for example in Turdidae birds [1], and is worthy of additional investigation.

The following steps can be recommended in research aiming to better understand the patterns of transmission of avian haemosporidian parasites at a study site. First, the prevalence and identity of *Haemoproteus* parasites should be determined using morphological and PCR-based methods in juvenile and adult birds. Secondly, one should use molecular methods to detect haemosporidian parasites in wild caught biting midges to provide data about links between parasites and possible vectors. Thirdly, it is necessary to prove that a given parasite lineage develops to the sporozoite stage in possible vectors [1, 14, 15]. Thus, a combination of microscopic, molecular and ecological approaches is needed in determining the natural vectors and patterns of parasite transmission. This methodology is essential for better understanding the epidemiology of avian haemoproteosis.

## Conclusions

*Culicoides impunctatus* is the likely natural vector of *H. pallidus* (hPFC1), *H*. *majoris* (hPARUS1), *H*. *motacillae* (hYWT1) and *H*. *balmorali* (hSFC9), and is susceptible to nine other species of avian haemoproteids. Phylogenetic analyses using partial *cytb* sequences is helpful in predicting possible vectors and some patterns of sporogonic development (morphology, rate of ookinete development), especially in closely related genetic lineages of *Haemoproteus* species. Morphological characters of ookinetes and sporozoites, as well as the rate of ookinete development, are markedly different in some *Haemoproteus* species, and these characters are worth more attention in the taxonomic research of haemoproteids at the species levels.

## Additional file

Additional file 1: Table S1. Morphometric parameters of ookinetes of four *Haemoproteus* species in the biting midge *Culicoides impunctatus*. Measurements are given in micrometres. **Table S2** Morphometric parameters of oocysts of four *Haemoproteus* species in the biting midge *Culicoides impunctatus*. Measurements are given in micrometres. **Table S3** Morphometric parameters of sporozoites of four *Haemoproteus* species in the biting midge *Culicoides impunctatus.* Measurements are given in micrometres. (XLS 36 kb)

## Abbreviations

dpi: Days post-infection
hpi: Hours post-infection
L/D: Light-dark
RH: Relative humidity

## References

[1] Valkiūnas G. Avian malaria parasites and other haemosporidia. Boca Raton, USA: CRC Press; 2005.

[2] Atkinson CT, Atkinson CT, Thomas NJ, Hunter BC (2008). Haemoproteus. Parasitic diseases of wild birds.

[3] Greiner EC (1975). Prevalence and potential vectors of *Haemoproteus* in Nebraska mourning doves. J Wildl Dis.

[4] White EM, Bennett GF. Avian Haemoproteidae. 9. Description of *Haemoproteus stellaris* n. sp. and a review of the haemoproteids of the swallow family Hirundinidae. Can J Zool. 1978;56:2110–6.

[5] Bensch S, Hellgren O, Pérez-Tris J (2009). A public database of malaria parasites and related haemosporidians in avian hosts based on mitochondrial cytochrome *b* lineages. Mol Ecol Resour.

[6] Ricklefs RE, Outlaw DC (2010). A molecular clock for malaria parasites. Science.

[7] Clark NJ, Clegg SM, Lima MR (2014). A review of global diversity in avian haemosporidians (*Plasmodium* and *Haemoproteus*: Haemosporida): new insights from molecular data. Int J Parasitol.

[8] Santiago-Alarcon D, Palinauskas V, Schaefer HM. Diptera vectors of avian haemosporidian parasites: untangling parasite life cycles and their taxonomy. Biol Rev Camb Philos Soc. 2012;87:928–64. 10.1111/j.1469-185X.2012.00234.x 22616880

[9] Bobeva A, Zehtindjiev P, Ilieva M, Dimitrov D, Mathis A, Bensch S (2015). Host preferences of ornithophilic biting midges of the genus *Culicoides* in the eastern Balkans. Med Vet Entomol.

[10] Kimura M, Darbro JM, Harrington LC (2010). Avian malaria parasites share congeneric mosquito vectors. J Parasitol.

[11] Valkiūnas G, Kazlauskienė R, Bernotienė R, Palinauskas V, Iezhova TA (2013). Abortive long-lasting sporogony of two *Haemoproteus* species (Haemosporida, Haemoproteidae) in the mosquito *Ochlerotatus cantans*, with perspectives on haemosporidian vector research. Parasitol Res.

[12] Valkiūnas G (2011). Haemosporidian vector research: marriage of molecular and microscopical approaches is essential. Mol Ecol.

[13] Kim KS, Tsuda Y, Sasaki T, Kobayashi M, Hirota Y (2009). Mosquito blood-meal analysis for avian malaria study in wild bird communities: laboratory verification and application to *Culex sasai* (Diptera: Culicidae) collected in Tokyo, Japan. Parasitol Res.

[14] Žiegytė R, Palinauskas V, Bernotienė R, Iezhova TA, Valkiūnas G (2014). *Haemoproteus minutus* and *Haemoproteus belopolskyi* (Haemoproteidae): complete sporogony in the biting midge *Culicoides impunctatus* (Ceratopogonidae), with implications on epidemiology of Haemoproteosis. Exp Parasitol.

[15] Bukauskaitė D, Žiegytė R, Palinauskas V, Iezhova TA, Dimitrov D, Ilgūnas M (2015). Biting midges (Culicoides, Diptera) transmit *Haemoproteus* parasites of owls: evidence from sporogony and molecular phylogeny. Parasit Vectors.

[16] Martínez-de la Puente J, Martínez J, Rivero-de Aguilar J, Herrero J, Merino S (2011). On the specificity of avian blood parasites: revealing specific and generalist relationships between haemosporidians and biting midges. Mol Ecol.

[17] Ferraguti M, Martinez-de la Puente J, Ruiz S, Soriguer R, Figuerola J (2013). On the study of the transmission network of blood parasites from SW Spain: diversity of avian haemosporidians in the biting midge *Culicoides circumscriptus* and wild birds. Parasit Vectors.

[18] Synek P, Munclinger P, Albrecht T, Votýpka J (2013). Avian haemosporidians in haematophagous insects in the Czech Republic. Parasitol Res.

[19] Tanigawa M, Sato Y, Ejiri H, Imura T, Chiba R, Yamamoto H (2013). Molecular identification of avian haemosporidia in wild birds and mosquitoes on Tsushima Island. Jpn J Vet Med Sci.

[20] Atkinson CT, Forrester DJ, Greiner EC (1988). Pathogenicity of *Haemoproteus meleagridis* (Haemosporina: Haemoproteidae) in experimentally infected domestic turkeys. J Parasitol.

[21] Earlé RA, Bastianello SS, Bennett GF, Krecek RC (1993). Histopathology and morphology of the tissue stages of *Haemoproteus columbae* causing mortality in Columbiformes. Avian Pathol.

[22] Cardona CJ, Ihejirika A, McClellan L (2002). *Haemoproteus lophortyx* infection in bobwhite quail. Avian Dis.

[23] Cannell BL, Krasnec KV, Campbell K, Jones HI, Miller RD, Stephens N (2013). The pathology and pathogenicity of a novel *Haemoproteus* spp. infection in wild little penguins (*Eudyptula minor*). Vet Parasitol.

[24] Tostes R, Martinele I, Vashist U, Castañon MC, Pinto Pde F, Daemon E, D’Agosto M (2015). Molecular characterization and biochemical and histopathological aspects of the parasitism of *Haemoproteus* spp. in southern caracaras (*Caracara plancus*). J Parasitol.

[25] Donovan TA, Schrenzel M, Tucker TA, Pessier AP, Stalis IH (2008). Hepatic hemorrhage, hemocoelom, and sudden death due to Haemoproteus infection in passerine birds: eleven cases. J Vet Diagn Investig.

[26] Olias PM, Wegelin W, Zenker S, Freter A, Gruber D, Klopfleisch R (2011). Avian malaria deaths in parrots. Eur Emerg Infect Dis.

[27] Valkiūnas G, Iezhova TA (2004). Detrimental effects of *Haemoproteus* infections on the survival of biting midge *Culicoides impunctatus* (Diptera: Ceratopogonidae). J Parasitol.

[28] Levin II, Parker PG (2014). Infection with *Haemoproteus iwa* affects vector movement in a hippoboscid fly-frigatebird system. Mol Ecol.

[29] Valkiūnas G, Kazlauskienė R, Bernotienė R, Bukauskaitė D, Palinauskas V, Iezhova TA (2014). *Haemoproteus* infections (Haemosporida, Haemoproteidae) kill bird-biting mosquitoes. Parasitol Res.

[30] Bukauskaitė D, Bernotienė R, Iezhova TA, Valkiūnas G (2016). Mechanisms of mortality in *Culicoides* biting midges due to *Haemoproteus* infection. Parasitology.

[31] Atkinson CT, van Riper III, C. Pathogenicity and epizootiology of avian haematozoa: *Plasmodium*, *Leucocytozoon*, and *Haemoproteus*. In: Loye, JE, Zuk, M, editors. Bird-parasite interactions: ecology, evolution, and behaviour. Oxford, UK: Oxford University Press; 1991. p. 19–48.

[32] Levin II, Zwiers P, Deem SL, Geest EA, Higashiguchi JM, Iezhova TA (2013). Multiple lineages of avian malaria parasites (*Plasmodium*) in the Galapagos Islands and evidence for arrival via migratory birds. Conserv Biol.

[33] Valkiūnas G, Liutkevičius G, Iezhova TA (2002). Complete development of three species of *Haemoproteus* (Haemosporida, Haemoproteidae) in the biting midge *Culicoides impunctatus* (Diptera, Ceratopogonidae). J Parasitol.

[34] Dimitrov D, Valkiūnas G, Zehtindjiev P, Ilieva M, Bensch S (2013). Molecular characterization of haemosporidian parasites (Haemosporida) in yellow wagtail (*Motacilla flava*), with description of in vitro ookinetes of *Haemoproteus motacillae*. Zootaxa.

[35] Bernotienė R, Valkiūnas G (2016). PCR detection of malaria parasites and related haemosporidians: the sensitive methodology in determining bird-biting insects. Malar J.

[36] Payevsky VA. Rybachy-type trap. In: Busse P, editor. Bird station manual. Gdansk (Poland): Gdansk University Press; 2000. p. 20–24.

[37] Sokolov LV, Vysotsky VG. [Ability for small range homing behaviour in pied flycatcher males (*Ficedula hуpoleuca*).] Zool Zhurnal. 1991;70:109–18. (In Russian).

[38] Hellgren O, Waldenstrom J, Bensch S (2004). A new PCR assay for simultaneous studies of *Leucocytozoon*, *Plasmodium*, and *Haemoproteus* from avian blood. J Parasitol.

[39] Valkiūnas G, Iezhova TA, Križanauskienė A, Palinauskas V, Bensch S (2008). A comparative analysis of microscopy and PCR-based detection methods for blood parasites. J Parasitol.

[40] Godfrey RD, Fedynich AM, Pence DB (1987). Quantification of hematozoa in blood smears. J Wildl Dis.

[41] Glukhova VM, Valkiūnas G. On the fauna and ecology of biting midges (Ceratopogonidae: *Culicoides*) in the Curonian spit, the methods of their collection from the birds and experimental infection with haemoproteids (Haemosporidia: Haemoproteidae). Ekologija. 1993;2:68–73.

[42] Liutkevičius G. The new data on the epidemiology of bird haemoproteids (Haemosporida: Haemoproteidae) on the Curonian Spit. Acta Zool Lithuan. 2000;2:72–7.

[43] Gutsevich AV. [The bloodsucking midges (Ceratopogonidae), in the fauna of the USSR. Dipteran insects.] Vol.3, part 5. Moscow: Nauka Press; 1973. (In Russian).

[44] Kazlauskienė R, Bernotienė R, Palinauskas V, Iezhova TA, Valkiūnas G (2013). *Plasmodium relictum* (lineages pSGS1 and pGRW11): complete synchronous sporogony in mosquitoes *Culex pipiens pipiens*. Exp Parasitol.

[45] Richardson DS, Jury FL, Blaakmeer K, Komdeur J, Burke T (2001). Parentage assignment and extra-group paternity in a cooperative breeder: the Seychelles warbler (*Acrocephalus sechellensis*). Mol Ecol.

[46] Bensch S, Stjenman M, Hasselquist D, Ostman O, Hansson B, Westerdahl H, Pinheiro RT (2000). Host specificity in avian blood parasites: a study of *Plasmodium* and *Haemoproteus* mitochondrial DNA amplified from birds. Proc R Soc B.

[47] Beadell JS, Gering E, Austin J, Dumbacher JP, Peirce MA, Pratt TK (2004). Prevalence and differential host-specificity of two avian blood parasite genera in the Australo-Papuan region. Mol Ecol.

[48] Folmer O, Black M, Hoeh W, Lutz R, Vrijenhoek R (1994). DNA primers for amplification of mitochondrial cytochrome *c* oxidase subunit I from diverse metazoan invertebrates. Mol Mar Biol Biotechnol.

[49] Ronquist F, Huelsenbeck JP (2003). MrBayes 3: Bayesian phylogenetic inference under mixed models. Bioinformatics.

[50] Nylander JAA. 2004. MrModeltest v2. Program distributed by the author. Software available at: https://github.com/nylander/MrModeltest2. Accessed 22 Aug 2017.

[51] Tamura K, Dudley J, Nei M, Kumar S (2007). MEGA4: molecular evolutionary genetics analysis (MEGA) software version 4.0. Mol Biol Evol.

[52] Fallis AM, Wood DM (1957). Biting midges (Diptera: Ceratopogonidae) as intermediate hosts for *Haemoproteus* of ducks. Can J Zool.

[53] Fallis AM, Bennett GF (1961). Sporogony of *Leucocytozoon* and *Haemoproteus* in simuliids and ceratopogonids and a revised classification of the Haemosporidiida. Can J Zool.

[54] Fallis AM, Bennett GF (1961). Ceratopogonidae as intermediate hosts for *Haemoproteus* and other parasites. Mosq News.

[55] Carpenter S, Groschup MH, Garros C, Felippe-Bauer ML, Purse B (2013). *Culicoides* biting midges, arboviruses and public health in Europe. Antivir Res.

[56] Blackwell AA, Mordue J, Mordue W (1994). Identification of bloodmeals of the Scottish biting midge, *Culicoides impunctatus*, by indirect enzyme-linked immunosorbent assay (ELISA). Med Vet Entomol.

[57] Žiegytė R, Bernotienė R, Palinauskas V, Valkiūnas G (2016). *Haemoproteus tartakovskyi* (Haemoproteidae): complete sporogony in *Culicoides nubeculosus* (Ceratopogonidae), with implications for avian haemoproteid experimental research. Exp Parasitol.

[58] Khan RA, Fallis AM (1971). A note on the sporogony of *Parahaemoproteus velans* (=*Haemoproteus velans* Coatney and Roudabush) (Haemosporidia: Haemoproteidae) in species of *Culicoides*. Can J Zool.

[59] Bennett GF, Fallis AM (1960). Blood parasites of birds in Algonquin Park, Canada, and a discussion of their transmission. Can J Zool.

[60] Valkiūnas G, Iezhova T. [Gametogenesis, zygote and ookinete of some species of bird haemoproteids (Haemosporidia: Haemoproteidae) formation in vitro.] Parazitologiya. 1993;27:19–35. (In Russian). 8474762

[61] Valkiūnas G, Iezhova T. [Peculiarities of the gametogenesis, zygote and ookinete of some species of bird haemoproteids (Haemosporidia: Haemoproteidae) formation in vitro.] Parazitologiya. 1994;28:36–47. (In Russian).

[62] Valkiūnas G, Iezhova T. [Peculiarities of the gametogenesis, zygote and ookinete of *Haemoproteus lanii* and *H. minutus* (Haemosporidia: Haemoproteidae) in vitro.] Parazitologiya. 1995;29:380–9. (In Russian).

[63] Nilsson E, Taubert H, Hellgren O, Huang X, Palinauskas V, Markovets MY (2016). Multiple cryptic species of sympatric generalists within the avian blood parasite *Haemoproteus majoris*. J Evol Biol.

[64] Hellgren O, Križanauskienė A, Valkiūnas G, Bensch S (2007). Diversity and phylogeny of mitochondrial cytochrome *b* lineages from six morphospecies of avian *Haemoproteus* (Haemosporida: Haemoproteidae). J Parasitol.

[65] Gilroy JJ, Anderson GQA, Grice PV, Vickery JA, Watts PN, Sutherland WJ (2009). Foraging habitat selection, diet and nestling condition in yellow wagtails *Motacilla flava* breeding on arable farmland. Bird Study.

[66] Kettle DS. The spatial distribution of *Culicoides impunctatus* Goet under woodland and moorland conditions and its flight range through woodland. Bull Entomol Res. 1951;42:239–91.

[67] Valkiūnas G, Križanauskienė A, Iezhova TA, Hellgren O, Bensch S. Molecular phylogenetic analysis of circumnuclear hemoproteids (Haemosporida: Haemoproteidae) of sylviid birds, with a description of *Haemoproteus parabelopolskyi *sp. nov. J Parasitol. 2007;93:680–7. 10.1645/GE-1102R.1 17626364

